# Supplementary material for: The Small RNA Universe of Capitella teleta
Source: Front Mol Biosci. 2022 Feb 25;9:802814. doi: 10.3389/fmolb.2022.802814 (PMC8915122; doi:10.3389/fmolb.2022.802814)
Supplement: Supplementary file 1 [file DataSheet1.ZIP › Supplement/known/CAPTEscaffold_70_6993.pdf]

Provisional ID	:	CAPTEscaffold_70_6993
Score total	:	11142269.5
Score for star read(s)	:	3.9
Score for read counts	:	11142260.8
Score for mfe	:	0.2
Score for randfold	:	1.6
Score for cons. seed	:	3
Total read count	:	21855076
Mature read count	:	21854914
Loop read count	:	0
Star read count	:	162

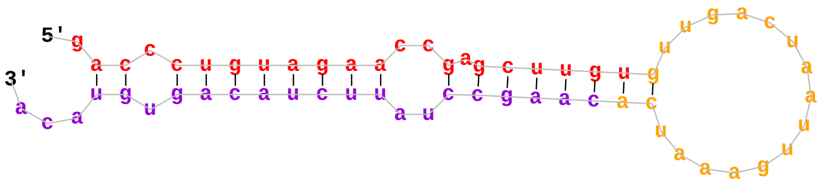

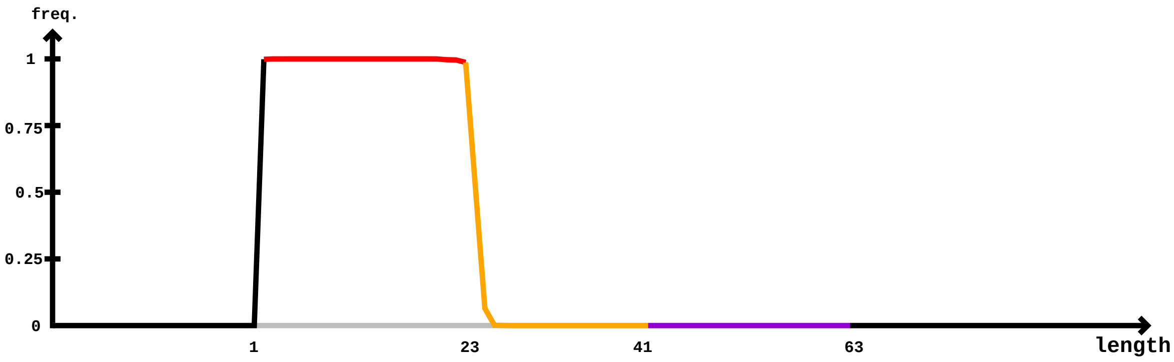

Mature Star

```
5'- ugaaagucacaaugcccauugacccuguagaaccgagcuuguguugacuaauugaaaucacaagccuauucuacaguguacaugguuauugcuaaaacucguccuucuucaa  -3'   obs
    ugaaagucacaaugcccauugacccuguagaaccgagcuuguguugacuaauugaaaucacaagccuauucuacaguguacaugguuauugcuaaaacucguccuucuucaa        exp
    ugaaagucacaaugcccauugacccuguagaaccgagcuuguguugacuaauugaaaucacaagccuauucuacaguguacaugguuauugcuaaaacucguccuucuucaa        known
    .........(((((.((((..((.((((((((..(.(((((((...............))))))))..)))))))).))..)))).))))).....................       reads   mm   sample
    ugaaagucacaaugcccauu............................................................................................       4       0    seq
    .................auugacccuguagaaccgagcuu.........................................................................       1       0    seq
    .................auCgacccuguagaaccgagcuugu......................................................................       205     1    seq
    .................Cuugacccuguagaaccgagcuugugu....................................................................       1       1    seq
    ..................uGgacccuguagaaccgagcu.........................................................................       2       1    seq
    ..................uCgacccuguagaaccgagcuug.......................................................................       1       1    seq
    ..................Cugacccuguagaaccgagcuugu......................................................................       2       1    seq
    ..................uugacccuguagaaccgagcuugu......................................................................       11      0    seq
    ..................uCgacccuguagaaccgagcuugu......................................................................       199     1    seq
    ..................uugacccuguagaaccgagcuugugu....................................................................       1       0    seq
    ..................uCgacccuguagaaccgagcuugugu....................................................................       1       1    seq
    ...................ugacccuguCgaaccgag..........................................................................       1       1    seq
    ...................uUacccuguagaaccgagc..........................................................................       1       1    seq
    ...................Cgacccuguagaaccgagcu.........................................................................       1       1    seq
    ...................uUacccuguagaaccgagcuug.......................................................................       1       1    seq
    ...................Cgacccuguagaaccgagcuug.......................................................................       20      1    seq
    ...................Ggacccuguagaaccgagcuugu......................................................................       1       1    seq
    ...................ugacccuguagaaUcgagcuugu......................................................................       1       1    seq
    ...................Agacccuguagaaccgagcuugu......................................................................       3       1    seq
    ...................ugacccuguagaaccgaAcuugu......................................................................       1       1    seq
    ...................ugacccuguagaaccgagcuugu......................................................................       94      0    seq
    ...................uAacccuguagaaccgagcuugu......................................................................       1       1    seq
    ...................Cgacccuguagaaccgagcuugu......................................................................       642     1    seq
    ...................uUacccuguagaaccgagcuugu......................................................................       130     1    seq
    ...................ugacccuguagaaccgagcuugug.....................................................................       2       0    seq
    ...................Cgacccuguagaaccgagcuugug.....................................................................       8       1    seq
    ...................uUacccuguagaaccgagcuugug.....................................................................       8       1    seq
    ...................Cgacccuguagaaccgagcuugugu....................................................................       3       1    seq
    ...................ugacccuguagaaccgagcuugugu....................................................................       2       0    seq
    ....................gaccUuguagaaccgagc..........................................................................       3       1    seq
    ....................gacccuguGgaaccgagc..........................................................................       2       1    seq
    ....................Aacccuguagaaccgagc..........................................................................       3       1    seq
```

```
                    Mature                                      Star
ugaaagucacaaugcccauugacccuguagaaccgagcuuguguugacuaauugaaaucacaagccuauucuacaguguacaugguuauugcuaaaacucguccuucuucaa
....................gacccugCagaaccgagc..........................................................................    1      1    seq
....................gacccuguagaaccgagA..........................................................................    593    1    seq
....................gacccugAagaaccgagc..........................................................................    1      1    seq
....................gacccuguagUaccgagc..........................................................................    1      1    seq
....................gacccuguagaaccgagU..........................................................................    39     1    seq
....................gNcccuguagaaccgagc..........................................................................    1      1    seq
....................gacccuguagaaccgagc..........................................................................    3295   0    seq
....................gacccuguagaaccgagG..........................................................................    2      1    seq
....................gacccuguagaUccgagc..........................................................................    1      1    seq
....................gaccAuguagaaccgagc..........................................................................    3      1    seq
....................gacccuguagaaccAagc..........................................................................    8      1    seq
....................gaAccuguagaaccgagc..........................................................................    1      1    seq
....................Nacccuguagaaccgagc..........................................................................    1      1    seq
....................gacUcuguagaaccgagc..........................................................................    2      1    seq
....................gGcccuguagaaccgagc..........................................................................    32     1    seq
....................gacccAguagaaccgagc..........................................................................    3      1    seq
....................Uacccuguagaaccgagc..........................................................................    7      1    seq
....................Cacccuguagaaccgagc..........................................................................    4      1    seq
....................gCcccuguagaaccgagc..........................................................................    1      1    seq
....................gacccuguagaaccgagcu.........................................................................    63201  0    seq
....................gacccuCuagaaccgagcu.........................................................................    7      1    seq
....................gacccuguagNaccgagcu.........................................................................    1      1    seq
....................gacccugAagaaccgagcu.........................................................................    37     1    seq
....................gacccuUuagaaccgagcu.........................................................................    1      1    seq
....................gacccuguagaacAgagcu.........................................................................    21     1    seq
....................gUcccuguagaaccgagcu.........................................................................    15     1    seq
....................gaNccuguagaaccgagcu.........................................................................    1      1    seq
....................gacccuguaUaaccgagcu.........................................................................    1      1    seq
....................gacccuguCgaaccgagcu.........................................................................    1      1    seq
....................gacccuguagaaccgagGu.........................................................................    24     1    seq
....................gacccuguagaCccgagcu.........................................................................    1      1    seq
....................gacccugCagaaccgagcu.........................................................................    1      1    seq
....................gNcccuguagaaccgagcu.........................................................................    4      1    seq
....................gacccuguagaaccgaUcu.........................................................................    5      1    seq
....................gacccuguagaaccgCgcu.........................................................................    3      1    seq
....................gCcccuguagaaccgagcu.........................................................................    15     1    seq
....................gacccuguagaaAcgagcu.........................................................................    17     1    seq
....................gacccuguagaaccgagcA.........................................................................    23     1    seq
....................gaccGuguagaaccgagcu.........................................................................    2      1    seq
....................gGcccuguagaaccgagcu.........................................................................    376    1    seq
....................gacccNguagaaccgagcu.........................................................................    2      1    seq
....................gacccuguGgaaccgagcu.........................................................................    11     1    seq
....................gacccuguagaaccUagcu.........................................................................    3      1    seq
....................gacccuguaCaaccgagcu.........................................................................    5      1    seq
....................gaccUuguagaaccgagcu.........................................................................    54     1    seq
....................gaGccuguagaaccgagcu.........................................................................    3      1    seq
....................gacccuguagaaccgGgcu.........................................................................    9      1    seq
....................gaUccuguagaaccgagcu.........................................................................    49     1    seq
....................gaAccuguagaaccgagcu.........................................................................    19     1    seq
....................gacccuguagaUccgagcu.........................................................................    5      1    seq
....................gacccuguagaaccgaCcu.........................................................................    2      1    seq
....................gacccCguagaaccgagcu.........................................................................    11     1    seq
....................gacccuguagaaccAagcu.........................................................................    90     1    seq
....................gacccuguagaGccgagcu.........................................................................    14     1    seq
....................gacccuguagaaccgagAu.........................................................................    7      1    seq
....................gacccuguagaaccgaAcu.........................................................................    1      1    seq
....................gacccuguagaacUgagcu.........................................................................    32     1    seq
....................gacccuguUgaaccgagcu.........................................................................    5      1    seq
....................gacccuguagaaccgUgcu.........................................................................    1      1    seq
....................gacccAguagaaccgagcu.........................................................................    32     1    seq
....................gacccuguagGaccgagcu.........................................................................    10     1    seq
....................gacccGguagaaccgagcu.........................................................................    1      1    seq
....................gacccuguagaaccgagcG.........................................................................    83     1    seq
....................Nacccuguagaaccgagcu.........................................................................    12     1    seq
....................gacccuguagaaccgagUu.........................................................................    4      1    seq
....................gacccuguagaaccgagcC.........................................................................    11     1    seq
....................gacccuguagaacGgagcu.........................................................................    2      1    seq
....................gacccuAuagaaccgagcu.........................................................................    13     1    seq
....................gacccuguaNaaccgagcu.........................................................................    1      1    seq
....................gacccuguagCaccgagcu.........................................................................    3      1    seq
```

```
                    Mature                                      Star
ugaaagucacaaugcccauugacccuguagaaccgagcuuguguugacuaauugaaaucacaagccuauucuacaguguacaugguuauugcuaaaacucguccuucuucaa
....................gacAcuguagaaccgagcu.........................................................................   16      1   seq
....................gacccugGagaaccgagcu.........................................................................   1       1   seq
....................gaccAuguagaaccgagcu.........................................................................   6       1   seq
....................Uacccuguagaaccgagcu.........................................................................   104     1   seq
....................gacccuguaAaaccgagcu.........................................................................   19      1   seq
....................gacccuguagaaccgaNcu.........................................................................   1       1   seq
....................gacccuguagaaGcgagcu.........................................................................   1       1   seq
....................gacUcuguagaaccgagcu.........................................................................   35      1   seq
....................gacccuguagaaUcgagcu.........................................................................   5       1   seq
....................Aacccuguagaaccgagcu.........................................................................   62      1   seq
....................Cacccuguagaaccgagcu.........................................................................   73      1   seq
....................gacccuguagUaccgagcu.........................................................................   1       1   seq
....................Aacccuguagaaccgagcuu........................................................................   26      1   seq
....................gacccuguagaaccgagcuu........................................................................   19044   0   seq
....................gacccuguagaaccgCgcuu........................................................................   1       1   seq
....................Nacccuguagaaccgagcuu........................................................................   6       1   seq
....................gacUcuguagaaccgagcuu........................................................................   7       1   seq
....................gacccuguaUaaccgagcuu........................................................................   2       1   seq
....................gacccuguagaaccUagcuu........................................................................   1       1   seq
....................gacccuguagaacGgagcuu........................................................................   2       1   seq
....................gaAccuguagaaccgagcuu........................................................................   5       1   seq
....................gacccuguagaGccgagcuu........................................................................   3       1   seq
....................gacccuguagaaccgagcCu........................................................................   4       1   seq
....................gacccuguagaacUgagcuu........................................................................   6       1   seq
....................gaccAuguagaaccgagcuu........................................................................   1       1   seq
....................Cacccuguagaaccgagcuu........................................................................   18      1   seq
....................gacccuguagaaccgagcuG........................................................................   2       1   seq
....................gacccuguagaaccgagAuu........................................................................   1       1   seq
....................gacccuCuagaaccgagcuu........................................................................   1       1   seq
....................gaGccuguagaaccgagcuu........................................................................   1       1   seq
....................gacccuguaCaaccgagcuu........................................................................   2       1   seq
....................gacccCguagaaccgagcuu........................................................................   2       1   seq
....................gacccuguagaacAgagcuu........................................................................   5       1   seq
....................gacccuguagaaccgagcuA........................................................................   26      1   seq
....................gacccuguagaaGcgagcuu........................................................................   1       1   seq
....................gacccugCagaaccgagcuu........................................................................   2       1   seq
....................gacccuguagaaccgagUuu........................................................................   1       1   seq
....................gacccuguagGaccgagcuu........................................................................   2       1   seq
....................gacccugAagaaccgagcuu........................................................................   10      1   seq
....................gacccuguagaaccCagcuu........................................................................   1       1   seq
....................gacccuAuagaaccgagcuu........................................................................   1       1   seq
....................gaccUuguagaaccgagcuu........................................................................   3       1   seq
....................gacccuguagaaccgaUcuu........................................................................   1       1   seq
....................gacccuguaAaaccgagcuu........................................................................   2       1   seq
....................Uacccuguagaaccgagcuu........................................................................   35      1   seq
....................gacccuguagaaccgagcAu........................................................................   1       1   seq
....................gacccuguagaaccgagcGu........................................................................   2       1   seq
....................gacccAguagaaccgagcuu........................................................................   13      1   seq
....................gacAcuguagaaccgagcuu........................................................................   5       1   seq
....................gacccuguagaaccgagGuu........................................................................   1       1   seq
....................gacccuguUgaaccgagcuu........................................................................   2       1   seq
....................gacccuguagaaccAagcuu........................................................................   37      1   seq
....................gacccuguagaaccgGgcuu........................................................................   1       1   seq
....................gacccuguagaaAcgagcuu........................................................................   5       1   seq
....................gaUccuguagaaccgagcuu........................................................................   2       1   seq
....................gGcccuguagaaccgagcuu........................................................................   129     1   seq
....................gacccuguagaaccgaCcuu........................................................................   1       1   seq
....................gacccuguagaaUcgagcuu........................................................................   1       1   seq
....................gacccuguGgaaccgagcuu........................................................................   8       1   seq
....................gCcccuguagaaccgagcuu........................................................................   16      1   seq
....................gacccuguagaaccgagcuC........................................................................   2       1   seq
....................gGcccuguagaaccgagcuug.......................................................................   1412    1   seq
....................gacccuguagaacNgagcuug.......................................................................   1       1   seq
....................gacUcuguagaaccgagcuug.......................................................................   75      1   seq
....................gacccuCuagaaccgagcuug.......................................................................   27      1   seq
....................gacccuguagaaccgagcAug.......................................................................   24      1   seq
....................gacccuguagaaccgUgcuug.......................................................................   6       1   seq
....................gacccuguagaaccgaNcuug.......................................................................   1       1   seq
....................gacccuguCgaaccgagcuug.......................................................................   4       1   seq
....................gacccuguagaaccgagcuAg.......................................................................   54      1   seq
```

```
                    Mature                                  Star
ugaaagucacaaugcccauugacccuguagaaccgagcuuguguugacuaauugaaaucacaagccuauucuacaguguacaugguuauugcuaaaacucguccuucuucaa
....................gacccuguagaaccgagcuuA.......................................................................	108	1	seq
....................gacccuAuagaaccgagcuug.......................................................................	23	1	seq
....................gacccGguagaaccgagcuug.......................................................................	8	1	seq
....................gacccuguaCaaccgagcuug.......................................................................	9	1	seq
....................gacccuguagaaccgagcuuC.......................................................................	9	1	seq
....................gaUccuguagaaccgagcuug.......................................................................	19	1	seq
....................gacccuguagaaccgagcCug.......................................................................	21	1	seq
....................gacccuguagaaAcgagcuug.......................................................................	36	1	seq
....................gacccuguagaGccgagcuug.......................................................................	33	1	seq
....................gacccuguUgaaccgagcuug.......................................................................	19	1	seq
....................gacccuguagCaccgagcuug.......................................................................	3	1	seq
....................gCcccuguagaaccgagcuug.......................................................................	63	1	seq
....................gacccuguagGaccgagcuug.......................................................................	11	1	seq
....................gacccuguagaaccgagAuug.......................................................................	21	1	seq
....................gaccAuguagaaccgagcuug.......................................................................	28	1	seq
....................gacccuguagaacAgagcuug.......................................................................	59	1	seq
....................gaGccuguagaaccgagcuug.......................................................................	5	1	seq
....................gNcccuguagaaccgagcuug.......................................................................	7	1	seq
....................gacccuguagaaccgaUcuug.......................................................................	76	1	seq
....................gacccuguaUaaccgagcuug.......................................................................	22	1	seq
....................gacAcuguagaaccgagcuug.......................................................................	66	1	seq
....................Uacccuguagaaccgagcuug.......................................................................	243	1	seq
....................gacccCguagaaccgagcuug.......................................................................	24	1	seq
....................gacccuguagaaGcgagcuug.......................................................................	5	1	seq
....................gacccuguagaaccgagcGug.......................................................................	15	1	seq
....................gacccuguagaaccUagcuug.......................................................................	13	1	seq
....................gacccuguagaaccgagcuuU.......................................................................	308	1	seq
....................gacccugCagaaccgagcuug.......................................................................	29	1	seq
....................Nacccuguagaaccgagcuug.......................................................................	47	1	seq
....................gaAccuguagaaccgagcuug.......................................................................	45	1	seq
....................gacccugGagaaccgagcuug.......................................................................	2	1	seq
....................gacccuguagaaccAagcuug.......................................................................	354	1	seq
....................gacccuguagaaccgagcuuN.......................................................................	1	1	seq
....................gacccuguagUaccgagcuug.......................................................................	17	1	seq
....................gacccuguagaaccgagUuug.......................................................................	89	1	seq
....................gacccuguagaaccgagGuug.......................................................................	14	1	seq
....................gaccUuguagaaccgagcuug.......................................................................	107	1	seq
....................gacccuguagaaccgagcuCg.......................................................................	29	1	seq
....................gacccuguagaaccgagcuug.......................................................................	192698	0	seq
....................gacccuguagaacGgagcuug.......................................................................	7	1	seq
....................gacccuguNgaaccgagcuug.......................................................................	1	1	seq
....................gacccuguGgaaccgagcuug.......................................................................	46	1	seq
....................Aacccuguagaaccgagcuug.......................................................................	184	1	seq
....................gUcccuguagaaccgagcuug.......................................................................	20	1	seq
....................gacccNguagaaccgagcuug.......................................................................	3	1	seq
....................gacccuguagaaUcgagcuug.......................................................................	18	1	seq
....................gacccuguagaaccgaCcuug.......................................................................	12	1	seq
....................gacccuguagaaccgaAcuug.......................................................................	8	1	seq
....................gaccGuguagaaccgagcuug.......................................................................	13	1	seq
....................Cacccuguagaaccgagcuug.......................................................................	182	1	seq
....................gacccuguagaaccgagcuGg.......................................................................	79	1	seq
....................gacccuguagaaccgNgcuug.......................................................................	1	1	seq
....................gacccuguagaacUgagcuug.......................................................................	61	1	seq
....................gacccAguagaaccgagcuug.......................................................................	111	1	seq
....................gacccugAagaaccgagcuug.......................................................................	92	1	seq
....................gacccuguaAaaccgagcuug.......................................................................	28	1	seq
....................gacccuguagaUccgagcuug.......................................................................	20	1	seq
....................gacccuguagaaccgCgcuug.......................................................................	6	1	seq
....................gacccuUuagaaccgagcuug.......................................................................	11	1	seq
....................gacGcuguagaaccgagcuug.......................................................................	5	1	seq
....................gacccuguagaCccgagcuug.......................................................................	3	1	seq
....................gacccuguagaaccCagcuug.......................................................................	5	1	seq
....................gacccuguaNaaccgagcuug.......................................................................	1	1	seq
....................gacccuguagaaccgGgcuug.......................................................................	33	1	seq
....................gacccuguagaaccgagcuugN......................................................................	42	1	seq
....................gacccuguagaaUcgagcuugu......................................................................	1835	1	seq
....................gacccuguagaaccgagcuAgu......................................................................	5001	1	seq
....................gacccuguagaaccgagcuugC......................................................................	1898	1	seq
....................gacccuguagaaccAagcuugu......................................................................	36263	1	seq
....................Cacccuguagaaccgagcuugu......................................................................	17680	1	seq
```

```
                    Mature                Star
ugaaagucacaaugcccauugacccuguagaaccgagcuuguguugacuaauugaaaucacaagccuauucuacaguguacaugguuauugcuaaaacucguccuucuucaa
....................gaccAuguagaaccgagcuugu......................................................................   3283      1   seq
....................Nacccuguagaaccgagcuugu......................................................................   3872      1   seq
....................gaGccuguagaaccgagcuugu......................................................................   534       1   seq
....................gacccuguagaaccgaUcuugu......................................................................   1639      1   seq
....................gacccuguagaaccgagcCugu......................................................................   1649      1   seq
....................gacccuguagUaccgagcuugu......................................................................   1424      1   seq
....................gacccuAuagaaccgagcuugu......................................................................   2866      1   seq
....................gacccuguagCaccgagcuugu......................................................................   262       1   seq
....................gacccNguagaaccgagcuugu......................................................................   455       1   seq
....................gacccuguagaaccgagcAugu......................................................................   2097      1   seq
....................gacccuguagaaccgagGuugu......................................................................   934       1   seq
....................gacccuguagaaccUagcuugu......................................................................   1300      1   seq
....................gacccuguaNaaccgagcuugu......................................................................   85        1   seq
....................gacccuguagaaccgagcuugG......................................................................   18874     1   seq
....................gacccuguagaaccgagcuugA......................................................................   11265     1   seq
....................gacccuNuagaaccgagcuugu......................................................................   9         1   seq
....................gacccuguagaaccgagAuugu......................................................................   2206      1   seq
....................gacccuguaAaaccgagcuugu......................................................................   3498      1   seq
....................gacccuguagaaccgagcGugu......................................................................   1485      1   seq
....................gacccuguagaaccgagcuCgu......................................................................   2023      1   seq
....................gUccuguagaaccgagcuugu.......................................................................   1812      1   seq
....................gacccuguagaaccgagcuuCu......................................................................   1328      1   seq
....................gacAcuguagaaccgagcuugu......................................................................   4889      1   seq
....................gacccuguagaaAcgagcuugu......................................................................   3343      1   seq
....................gacccuguagaaccgaAcuugu......................................................................   924       1   seq
....................gacUcuguagaaccgagcuugu......................................................................   7930      1   seq
....................gacccuguagaaccgagUuugu......................................................................   1437      1   seq
....................gacccuguagaacNgagcuugu......................................................................   28        1   seq
....................gacccuguagaaccgagcuugu......................................................................   18346071  0   seq
....................gacccuguNgaaccgagcuugu......................................................................   25        1   seq
....................gacccuguagaaccgGgcuugu......................................................................   3052      1   seq
....................gCcccuguagaaccgagcuugu......................................................................   5624      1   seq
....................gaAccuguagaaccgagcuugu......................................................................   4747      1   seq
....................gacccuguagaUccgagcuugu......................................................................   1956      1   seq
....................gacccuguagaaccgagNuugu......................................................................   1         1   seq
....................gaNccuguagaaccgagcuugu......................................................................   57        1   seq
....................gacccuguagaaccCagcuugu......................................................................   663       1   seq
....................gacccGguagaaccgagcuugu......................................................................   498       1   seq
....................gacccuguagaacAgagcuugu......................................................................   4340      1   seq
....................gacccuguagGaccgagcuugu......................................................................   2088      1   seq
....................gacccugAagaaccgagcuugu......................................................................   8131      1   seq
....................gaUccuguagaaccgagcuugu......................................................................   1736      1   seq
....................gacccuguagaaccgNgcuugu......................................................................   22        1   seq
....................gacccuguagaaccgUgcuugu......................................................................   877       1   seq
....................gacccAguagaaccgagcuugu......................................................................   9609      1   seq
....................gacccugGagaaccgagcuugu......................................................................   328       1   seq
....................gacccuguaUaaccgagcuugu......................................................................   1111      1   seq
....................gacccuguagaaccgagcuuNu......................................................................   93        1   seq
....................gaccUuguagaaccgagcuugu......................................................................   11313     1   seq
....................gaccGuguagaaccgagcuugu......................................................................   644       1   seq
....................gacccuguagaaccgagcuuUu......................................................................   875       1   seq
....................gacccuguagNaccgagcuugu......................................................................   30        1   seq
....................gacccuguUgaaccgagcuugu......................................................................   1542      1   seq
....................gacccuguagaaccgagcuNgu......................................................................   20        1   seq
....................gacccugCagaaccgagcuugu......................................................................   2209      1   seq
....................gacccuguagaaccgaNcuugu......................................................................   24        1   seq
....................gacccuguagaaccgagcuGgu......................................................................   1064      1   seq
....................gacccuguagaaGcgagcuugu......................................................................   714       1   seq
....................gacccuguGgaaccgagcuugu......................................................................   3630      1   seq
....................gacccuguaCaaccgagcuugu......................................................................   966       1   seq
....................gacccuguagaaccgCgcuugu......................................................................   404       1   seq
....................gacccuguagaCccgagcuugu......................................................................   152       1   seq
....................gGcccuguagaaccgagcuugu......................................................................   116160    1   seq
....................gacccuguagaaccNagcuugu......................................................................   1         1   seq
....................Uacccuguagaaccgagcuugu......................................................................   30409     1   seq
....................gacccuguagaaccgaCcuugu......................................................................   533       1   seq
....................gacccuguCgaaccgagcuugu......................................................................   189       1   seq
....................gacccuguagaacGgagcuugu......................................................................   409       1   seq
....................gaccNuguagaaccgagcuugu......................................................................   6         1   seq
....................gacccuguagaacUgagcuugu......................................................................   7926      1   seq
```

```
                    Mature                                    Star
ugaaagucacaaugcccauugacccuguagaaccgagcuuguguugacuaauugaaaucacaagccuauucuacaguguacaugguuauugcuaaaacucguccuucuucaa
....................gacccuguagaNccgagcuugu......................................................................   2        1   seq
....................Aacccuguagaaccgagcuugu......................................................................   20043    1   seq
....................gacccugNagaaccgagcuugu......................................................................   4        1   seq
....................gacccuguagaGccgagcuugu......................................................................   3533     1   seq
....................gacccuguagaaccgagcuuAu......................................................................   1941     1   seq
....................gacccuUuagaaccgagcuugu......................................................................   1017     1   seq
....................gacccuguagaaccgagcNugu......................................................................   21       1   seq
....................gacccuCuagaaccgagcuugu......................................................................   1351     1   seq
....................gacccCguagaaccgagcuugu......................................................................   2618     1   seq
....................gNcccuguagaaccgagcuugu......................................................................   706      1   seq
....................gacGcuguagaaccgagcuugu......................................................................   644      1   seq
....................gacccugGagaaccgagcuugug.....................................................................   16       1   seq
....................gCcccuguagaaccgagcuugug.....................................................................   467      1   seq
....................gacccugCagaaccgagcuugug.....................................................................   134      1   seq
....................gacccuguagaaccgagcuugGg.....................................................................   393      1   seq
....................gacccAguagaaccgagcuugug.....................................................................   723      1   seq
....................gacccuguagaCccgagcuugug.....................................................................   10       1   seq
....................gacccuguagaaAcgagcuugug.....................................................................   293      1   seq
....................gacccuguagaaccgagcuugAg.....................................................................   652      1   seq
....................gacccuguagGaccgagcuugug.....................................................................   154      1   seq
....................gacccuguagUaccgagcuugug.....................................................................   101      1   seq
....................gUcccuguagaaccgagcuugug.....................................................................   128      1   seq
....................gacccuguagaaccgagcAugug.....................................................................   161      1   seq
....................gacccuguaAaaccgagcuugug.....................................................................   283      1   seq
....................Aacccuguagaaccgagcuugug.....................................................................   1512     1   seq
....................gacccuguagaaccgagcuuUug.....................................................................   42       1   seq
....................gacccuguagaaccgaAcuugug.....................................................................   58       1   seq
....................gGcccuguagaaccgagcuugug.....................................................................   10291    1   seq
....................gaccAuguagaaccgagcuugug.....................................................................   252      1   seq
....................gacccuguagaaccgUgcuugug.....................................................................   48       1   seq
....................gacccuguaUaaccgagcuugug.....................................................................   79       1   seq
....................gacccuNuagaaccgagcuugug.....................................................................   3        1   seq
....................gaccNuguagaaccgagcuugug.....................................................................   1        1   seq
....................gacccuguagaacUgagcuugug.....................................................................   587      1   seq
....................gacccuguagaaccgaUcuugug.....................................................................   195      1   seq
....................gacccuguagaaccgGgcuugug.....................................................................   244      1   seq
....................gacccuguagaaccgagcuuguC.....................................................................   140      1   seq
....................gacccuguagaacNgagcuugug.....................................................................   1        1   seq
....................gacccuguagaaccgaCcuugug.....................................................................   114      1   seq
....................gacccGguagaaccgagcuugug.....................................................................   23       1   seq
....................gacccuguUgaaccgagcuugug.....................................................................   117      1   seq
....................gacccuguagaaccgaNcuugug.....................................................................   5        1   seq
....................gacccuguagaaccgagcuuguA.....................................................................   5283     1   seq
....................gacccuguagaaccgNgcuugug.....................................................................   3        1   seq
....................gaAccuguagaaccgagcuugug.....................................................................   357      1   seq
....................gacccuguagaaccgagcuAgug.....................................................................   345      1   seq
....................gaccGuguagaaccgagcuugug.....................................................................   46       1   seq
....................gNcccuguagaaccgagcuugug.....................................................................   41       1   seq
....................Uacccuguagaaccgagcuugug.....................................................................   1573     1   seq
....................gacccuguagaaccgagcGugug.....................................................................   124      1   seq
....................gacccuguagaaccgagcuCgug.....................................................................   125      1   seq
....................gacccuguagaaccgagAuugug.....................................................................   178      1   seq
....................gacAcuguagaaccgagcuugug.....................................................................   397      1   seq
....................gacUcuguagaaccgagcuugug.....................................................................   528      1   seq
....................gacccuguNgaaccgagcuugug.....................................................................   2        1   seq
....................gacccuguagNaccgagcuugug.....................................................................   3        1   seq
....................gacccuUuagaaccgagcuugug.....................................................................   75       1   seq
....................gacccuguagaaccgagcuuguU.....................................................................   54510    1   seq
....................gacccuguagaaccgagcuugug.....................................................................   1290040  0   seq
....................gacccuguagCaccgagcuugug.....................................................................   15       1   seq
....................gacccuguagaaUcgagcuugug.....................................................................   88       1   seq
....................gacccuAuagaaccgagcuugug.....................................................................   202      1   seq
....................gacccuguGgaaccgagcuugug.....................................................................   267      1   seq
....................gacccuguagaaccgagcuGgug.....................................................................   66       1   seq
....................gacccuguagaaccgagcuNgug.....................................................................   3        1   seq
....................gacccuguagaacGgagcuugug.....................................................................   39       1   seq
....................gacccuguagaaccCagcuugug.....................................................................   50       1   seq
....................gaNccuguagaaccgagcuugug.....................................................................   2        1   seq
....................gacccuguagaaccgagUuugug.....................................................................   72       1   seq
....................gacccNguagaaccgagcuugug.....................................................................   32       1   seq
```

```
                    Mature                                      Star
ugaaagucacaaugcccauugacccuguagaaccgagcuuguguugacuaauugaaaucacaagccuauucuacaguguacaugguuauugcuaaaacucguccuucuucaa
....................gacccuguagaaccgagcuugNg.....................................................................  4      1  seq
....................gacccuguagaaGcgagcuugug.....................................................................  44     1  seq
....................gacccuguagaaccUagcuugug.....................................................................  75     1  seq
....................gacccuguagaaccgagcuuAug.....................................................................  173    1  seq
....................gacccuguagaaccgagcuuguN.....................................................................  46     1  seq
....................gaGccuguagaaccgagcuugug.....................................................................  36     1  seq
....................gacccCguagaaccgagcuugug.....................................................................  140    1  seq
....................gacccugAagaaccgagcuugug.....................................................................  589    1  seq
....................Cacccuguagaaccgagcuugug.....................................................................  1069   1  seq
....................gacccuguagaaccgCgcuugug.....................................................................  27     1  seq
....................gaUccuguagaaccgagcuugug.....................................................................  131    1  seq
....................gacccuguaCaaccgagcuugug.....................................................................  64     1  seq
....................gacccuguagaaccgagcNugug.....................................................................  2      1  seq
....................gacccuguagaaccgagcuuNug.....................................................................  14     1  seq
....................gacccuguagaaccAagcuugug.....................................................................  2975   1  seq
....................gacccuguagaGccgagcuugug.....................................................................  263    1  seq
....................gacccuguagaaccgagcuugCg.....................................................................  155    1  seq
....................gacccuCuagaaccgagcuugug.....................................................................  105    1  seq
....................gacccuguagaaccgagGuugug.....................................................................  65     1  seq
....................gacccuguCgaaccgagcuugug.....................................................................  10     1  seq
....................gacccuguagaacAgagcuugug.....................................................................  335    1  seq
....................gacccuguaNaaccgagcuugug.....................................................................  6      1  seq
....................gacccuguagaUccgagcuugug.....................................................................  133    1  seq
....................gaccUuguagaaccgagcuugug.....................................................................  727    1  seq
....................gacccuguagaaccgagcuuCug.....................................................................  100    1  seq
....................gacGcuguagaaccgagcuugug.....................................................................  54     1  seq
....................gacccuguagaaccgagcCugug.....................................................................  97     1  seq
....................Nacccuguagaaccgagcuugug.....................................................................  249    1  seq
....................gacccuguaCaaccgagcuugugu....................................................................  42     1  seq
....................gacccuguagaNccgagcuugugu....................................................................  1      1  seq
....................gacccuAuagaaccgagcuugugu....................................................................  140    1  seq
....................gacccuguagaaccgagcuugugC....................................................................  1604   1  seq
....................gaccNuguagaaccgagcuugugu....................................................................  1      1  seq
....................gacccuguagaaccgagcuuguAu....................................................................  1258   1  seq
....................gacccuguagCaccgagcuugugu....................................................................  18     1  seq
....................gacccuguagaaccgagcGugugu....................................................................  82     1  seq
....................gacccuguaUaaccgagcuugugu....................................................................  56     1  seq
....................gacccuguaNaaccgagcuugugu....................................................................  6      1  seq
....................gUcccuguagaaccgagcuugugu....................................................................  92     1  seq
....................gacccuCuagaaccgagcuugugu....................................................................  59     1  seq
....................gacccuguagaaccgagcuuguCu....................................................................  51     1  seq
....................gacccuguagaaccgagcuugugG....................................................................  63287  1  seq
....................gacccuguagaaccgagcuGgugu....................................................................  44     1  seq
....................gacccuguagaaccCagcuugugu....................................................................  22     1  seq
....................gacccNguagaaccgagcuugugu....................................................................  17     1  seq
....................gacccuguagaaccgagcuuguNu....................................................................  11     1  seq
....................gaccUuguagaaccgagcuugugu....................................................................  519    1  seq
....................gacccAguagaaccgagcuugugu....................................................................  473    1  seq
....................gaAccuguagaaccgagcuugugu....................................................................  233    1  seq
....................gacccuguagaaccgagAuugugu....................................................................  90     1  seq
....................gacccCguagaaccgagcuugugu....................................................................  109    1  seq
....................gNccuguagaaccgagcuugugu.....................................................................  30     1  seq
....................gacccuguagaaGcgagcuugugu....................................................................  34     1  seq
....................gacccuguagaCccgagcuugugu....................................................................  7      1  seq
....................gacccuguagaacNgagcuugugu....................................................................  2      1  seq
....................gacccuguagaaccgagcuuguUu....................................................................  5922   1  seq
....................gacccugCagaaccgagcuugugu....................................................................  105    1  seq
....................Cacccuguagaaccgagcuugugu....................................................................  757    1  seq
....................gacccuguagaaccgGgcuugugu....................................................................  123    1  seq
....................gacccugGagaaccgagcuugugu....................................................................  15     1  seq
....................gacAcuguagaaccgagcuugugu....................................................................  216    1  seq
....................gacccuguUgaaccgagcuugugu....................................................................  58     1  seq
....................gacccuguagaaccgagcuuNugu....................................................................  4      1  seq
....................gacccuguagaaccgagcuugAgu....................................................................  417    1  seq
....................gacccuguaAaaccgagcuugugu....................................................................  156    1  seq
....................gacccuguagaaccgagcuCgugu....................................................................  87     1  seq
....................gaUccuguagaaccgagcuugugu....................................................................  125    1  seq
....................gacccuguagaaccgagGuugugu....................................................................  49     1  seq
....................gacccuguagaaccgUgcuugugu....................................................................  23     1  seq
....................gacccuguagaaccUagcuugugu....................................................................  59     1  seq
```

```
                    Mature                                        Star
ugaaagucacaaugcccauugacccuguagaaccgagcuuguguugacuaauugaaaucacaagccuauucuacaguguacaugguuauugcuaaaacucguccuucuucaa
....................gacccuguagaaccgaAcuugugu....................................................................	40	1	seq
....................Uacccuguagaaccgagcuugugu....................................................................	1240	1	seq
....................gacccuguagaaccgaCcuugugu....................................................................	16	1	seq
....................gacccuguagaaccgagcuugGgu....................................................................	52	1	seq
....................Nacccuguagaaccgagcuugugu....................................................................	186	1	seq
....................gacccugAagaaccgagcuugugu....................................................................	400	1	seq
....................gacccuguagaaccgagcuAgugu....................................................................	226	1	seq
....................gaGccuguagaaccgagcuugugu....................................................................	23	1	seq
....................gacccuguagGaccgagcuugugu....................................................................	96	1	seq
....................gacccuNuagaaccgagcuugugu....................................................................	1	1	seq
....................gGcccuguagaaccgagcuugugu....................................................................	5563	1	seq
....................gacccuguagaacGgagcuugugu....................................................................	17	1	seq
....................gaccGuguagaaccgagcuugugu....................................................................	24	1	seq
....................gacccuguagaaccgagcuuAugu....................................................................	78	1	seq
....................gacccuguagaUccgagcuugugu....................................................................	90	1	seq
....................gacccuguagaaccgagcuugCgu....................................................................	84	1	seq
....................gacccGguagaaccgagcuugugu....................................................................	19	1	seq
....................gacccuguagUaccgagcuugugu....................................................................	70	1	seq
....................gacccuguagaaccgagcuugugA....................................................................	445772	1	seq
....................gaccAuguagaaccgagcuugugu....................................................................	165	1	seq
....................gacccuguagaaccgCgcuugugu....................................................................	16	1	seq
....................gacccuguagaaccgagcuugugN....................................................................	8	1	seq
....................gacccuguagaGccgagcuugugu....................................................................	171	1	seq
....................gacccuguagaaAcgagcuugugu....................................................................	165	1	seq
....................gacccuguagaaccgaUcuugugu....................................................................	66	1	seq
....................gacccuguCgaaccgagcuugugu....................................................................	15	1	seq
....................gacGcuguagaaccgagcuugugu....................................................................	22	1	seq
....................Aacccuguagaaccgagcuugugu....................................................................	801	1	seq
....................gacccuguagaaccgagcuugugu....................................................................	856548	0	seq
....................gacUcuguagaaccgagcuugugu....................................................................	377	1	seq
....................gacccuguGgaaccgagcuugugu....................................................................	168	1	seq
....................gacccuguagaacAgagcuugugu....................................................................	210	1	seq
....................gacccuguagaaUcgagcuugugu....................................................................	71	1	seq
....................gacccuguagaaccgagcuuUugu....................................................................	27	1	seq
....................gacccuguagaaccgNgcuugugu....................................................................	1	1	seq
....................gacccuUuagaaccgagcuugugu....................................................................	49	1	seq
....................gCcccuguagaaccgagcuugugu....................................................................	243	1	seq
....................gacccuguagaaccgagUuugugu....................................................................	78	1	seq
....................gacccuguagaaccgagcuuCugu....................................................................	57	1	seq
....................gacccuguagaacUgagcuugugu....................................................................	406	1	seq
....................gacccuguagaaccgagcCugugu....................................................................	86	1	seq
....................gacccuguagaaccAagcuugugu....................................................................	1259	1	seq
....................gaNccuguagaaccgagcuugugu....................................................................	4	1	seq
....................gacccuguagaaccgagcAugugu....................................................................	96	1	seq
....................gacAcuguagaaccgagcuuguguu...................................................................	4	1	seq
....................gaGccuguagaaccgagcuuguguu...................................................................	1	1	seq
....................gacccuguagaaccgagGuuguguu...................................................................	1	1	seq
....................gacccuguagaaccgagUuuguguu...................................................................	2	1	seq
....................gacccuguagaaccgagcuuguAuu...................................................................	34	1	seq
....................gacccuguUgaaccgagcuuguguu...................................................................	2	1	seq
....................gacccuguagaaccgagcuuCuguu...................................................................	1	1	seq
....................gacccuCuagaaccgagcuuguguu...................................................................	1	1	seq
....................gaccGuguagaaccgagcuuguguu...................................................................	1	1	seq
....................gacccuguagaaccgagcuuguguC...................................................................	9	1	seq
....................gacccGguagaaccgagcuuguguu...................................................................	1	1	seq
....................gacccugCagaaccgagcuuguguu...................................................................	2	1	seq
....................gacccuguagaaccgagcuuguUuu...................................................................	506	1	seq
....................gacccuguagaaccgagcuugugAu...................................................................	3617	1	seq
....................gaAccuguagaaccgagcuuguguu...................................................................	4	1	seq
....................gacccuguagaaAcgagcuuguguu...................................................................	1	1	seq
....................gacccuguGgaaccgagcuuguguu...................................................................	2	1	seq
....................gacccuguagaacAgagcuuguguu...................................................................	6	1	seq
....................gacccuNuagaaccgagcuuguguu...................................................................	1	1	seq
....................gaUccuguagaaccgagcuuguguu...................................................................	2	1	seq
....................Aacccuguagaaccgagcuuguguu...................................................................	13	1	seq
....................gacccuguagaaccgagcuuguguA...................................................................	484	1	seq
....................Nacccuguagaaccgagcuuguguu...................................................................	1	1	seq
....................gacccuguagaaccgagcuuguguG...................................................................	93	1	seq
....................gacccuguagGaccgagcuuguguu...................................................................	2	1	seq
....................gacUcuguagaaccgagcuuguguu...................................................................	6	1	seq
```

```
                    Mature                                   Star
ugaaagucacaaugcccauugacccuguagaaccgagcuuguguugacuaauugaaaucacaagccuauucuacaguguacaugguuauugcuaaaacucguccuucuucaa
....................gacccuguagaaccgagcuuguguN...................................................................    1      1     seq
....................gaccUuguagaaccgagcuuguguu...................................................................    5      1     seq
....................gacccuguagaaccgagcuugAguu...................................................................    11     1     seq
....................gacccuguagaGccgagcuuguguu...................................................................    3      1     seq
....................gacccuguagaaccCagcuuguguu...................................................................    1      1     seq
....................gacccuguagaaccgagcuAguguu...................................................................    4      1     seq
....................gaccAuguagaaccgagcuuguguu...................................................................    1      1     seq
....................gacccuguagaaUcgagcuuguguu...................................................................    2      1     seq
....................gacccuguaAaaccgagcuuguguu...................................................................    3      1     seq
....................gacccuguagaaccgagcuuAuguu...................................................................    2      1     seq
....................gacccuguagaaccAagcuuguguu...................................................................    17     1     seq
....................gacccuguagaaccgagcuGguguu...................................................................    2      1     seq
....................gacccuAuagaaccgagcuuguguu...................................................................    1      1     seq
....................gacccuguagaaccgagcuuguguu...................................................................    13179  0     seq
....................gacccAguagaaccgagcuuguguu...................................................................    9      1     seq
....................gacccCguagaaccgagcuuguguu...................................................................    2      1     seq
....................Uacccuguagaaccgagcuuguguu...................................................................    21     1     seq
....................gacccuguagaaccgagcuugCguu...................................................................    4      1     seq
....................gGcccuguagaaccgagcuuguguu...................................................................    84     1     seq
....................gacccuguagaaccgagcuugugGu...................................................................    6      1     seq
....................gacccuguagaaccgagcAuguguu...................................................................    1      1     seq
....................gacccuguagaaccgagcGuguguu...................................................................    1      1     seq
....................gacccuUuagaaccgagcuuguguu...................................................................    1      1     seq
....................Cacccuguagaaccgagcuuguguu...................................................................    14     1     seq
....................gacccuguagaaccgagcuugugCu...................................................................    39     1     seq
....................gCcccuguagaaccgagcuuguguu...................................................................    4      1     seq
....................gacccuguagaaccgGgcuuguguu...................................................................    1      1     seq
....................gacccuguagaacUgagcuuguguu...................................................................    6      1     seq
....................gacccuguagaaccgaCcuuguguu...................................................................    1      1     seq
....................gacccuguagaaccgaUcuuguguu...................................................................    2      1     seq
....................gacccuguagaaccgUgcuuguguu...................................................................    1      1     seq
....................gacccugAagaaccgagcuuguguu...................................................................    4      1     seq
....................gacccuguagaaccgagcuuguguAg..................................................................    1      1     seq
....................gacccuguagaaccgagcuuguguGg..................................................................    2      1     seq
....................gacccuguagaaccgagcuuguguuC..................................................................    7      1     seq
....................gacccuguagaaccgagcuuguguuA..................................................................    141    1     seq
....................gacccuguagaaccgagcuuguguug..................................................................    1      0     seq
....................gacccuguagaaccgagcuuguguuU..................................................................    3252   1     seq
....................gacccuguagaaccgagcuuguguugacu...............................................................    3      0     seq
.....................acccAguagaaccgagcu.........................................................................    1      1     seq
.....................acccuguagaaccgagcu.........................................................................    81     0     seq
.....................aAccuguagaaccgagcu.........................................................................    2      1     seq
.....................Gcccuguagaaccgagcu.........................................................................    4      1     seq
.....................acAcuguagaaccgagcuu........................................................................    1      1     seq
.....................acccuguagaaccgagcuu........................................................................    19     0     seq
.....................Gcccuguagaaccgagcuug.......................................................................    4      1     seq
.....................aAccuguagaaccgagcuug.......................................................................    1      1     seq
.....................acccuguagaaccgagcuuU.......................................................................    1      1     seq
.....................acccuguagaaccgagcuug.......................................................................    119    0     seq
.....................Gcccuguagaaccgagcuugu......................................................................    301    1     seq
.....................acccuguagaaccgagcuugG......................................................................    17     1     seq
.....................aUccuguagaaccgagcuugu......................................................................    4      1     seq
.....................acccugAagaaccgagcuugu......................................................................    19     1     seq
.....................acccuguaUaaccgagcuugu......................................................................    1      1     seq
.....................accUuguagaaccgagcuugu......................................................................    15     1     seq
.....................acccuUuagaaccgagcuugu......................................................................    3      1     seq
.....................acccuAuagaaccgagcuugu......................................................................    3      1     seq
.....................acccuguagaacGgagcuugu......................................................................    1      1     seq
.....................acccuguagUaccgagcuugu......................................................................    2      1     seq
.....................acccuguGgaaccgagcuugu......................................................................    1      1     seq
.....................acccuguagaacAgagcuugu......................................................................    19     1     seq
.....................acNcuguagaaccgagcuugu......................................................................    1      1     seq
.....................acccuguagaaccgGgcuugu......................................................................    3      1     seq
.....................acccugGagaaccgagcuugu......................................................................    2      1     seq
.....................Ccccuguagaaccgagcuugu......................................................................    26     1     seq
.....................acccuCuagaaccgagcuugu......................................................................    1      1     seq
.....................acccuguagaUccgagcuugu......................................................................    4      1     seq
.....................acccuguagaGccgagcuugu......................................................................    46     1     seq
.....................acccuguagaaccgagGuugu......................................................................    1      1     seq
.....................acccuguagaaccgaAcuugu......................................................................    2      1     seq
```

```
                    Mature                                  Star
ugaaagucacaaugcccauugacccuguagaaccgagcuuguguugacuaauugaaaucacaagccuauucuacaguguacaugguuauugcuaaaacucguccuucuucaa
.....................Ncccuguagaaccgagcuugu......................................................................    4      1   seq
.....................acccuguagaaccgUgcuugu......................................................................    1      1   seq
.....................aNccuguagaaccgagcuugu......................................................................    1      1   seq
.....................accAuguagaaccgagcuugu......................................................................    6      1   seq
.....................Ucccuguagaaccgagcuugu......................................................................    4      1   seq
.....................acccuguagaaccUagcuugu......................................................................    3      1   seq
.....................acccuguagaaccgagcuugu......................................................................    20886  0   seq
.....................acccuguagaaccgagcuCgu......................................................................    4      1   seq
.....................acccuguagaaccAagcuugu......................................................................    77     1   seq
.....................acccuguaAaaccgagcuugu......................................................................    4      1   seq
.....................acccuguUgaaccgagcuugu......................................................................    5      1   seq
.....................acccuguagaaccgagUuugu......................................................................    1      1   seq
.....................acccuguagGaccgagcuugu......................................................................    3      1   seq
.....................acccuguagaaccgagcuuNu......................................................................    1      1   seq
.....................acUcuguagaaccgagcuugu......................................................................    6      1   seq
.....................acccuguagaaccgagcAugu......................................................................    5      1   seq
.....................acccCguagaaccgagcuugu......................................................................    2      1   seq
.....................acccuguagaaccgagcuugC......................................................................    11     1   seq
.....................acccAguagaaccgagcuugu......................................................................    75     1   seq
.....................acAcuguagaaccgagcuugu......................................................................    9      1   seq
.....................acccuguagaaccgagAuugu......................................................................    3      1   seq
.....................acccuguagaaccgagcuugA......................................................................    14     1   seq
.....................acccuguagaacUgagcuugu......................................................................    11     1   seq
.....................acccuguagaaccgagcuAgu......................................................................    8      1   seq
.....................accGuguagaaccgagcuugu......................................................................    2      1   seq
.....................acccuguaCaaccgagcuugu......................................................................    1      1   seq
.....................acccuguagaaccgagcuuCu......................................................................    2      1   seq
.....................acccuguagaaAcgagcuugu......................................................................    9      1   seq
.....................aAccuguagaaccgagcuugu......................................................................    90     1   seq
.....................acccuguagaaccgagcuuAu......................................................................    2      1   seq
.....................acccuNuagaaccgagcuugu......................................................................    1      1   seq
.....................acccuAuagaaccgagcuugug.....................................................................    1      1   seq
.....................acccuguagaaccgagAuugug.....................................................................    1      1   seq
.....................acccuguagaaccgagcuugug.....................................................................    1106   0   seq
.....................acUcuguagaaccgagcuugug.....................................................................    1      1   seq
.....................acccuguagaaccgagcuuguA.....................................................................    86     1   seq
.....................Gcccuguagaaccgagcuugug.....................................................................    24     1   seq
.....................acccuguagaaccAagcuugug.....................................................................    6      1   seq
.....................acccuguagaaccgagcuuguC.....................................................................    3      1   seq
.....................aAccuguagaaccgagcuugug.....................................................................    5      1   seq
.....................acccuguagaaccgagcuugAg.....................................................................    2      1   seq
.....................acccugAagaaccgagcuugug.....................................................................    1      1   seq
.....................acccugCagaaccgagcuugug.....................................................................    1      1   seq
.....................acccuguagaaccgGgcuugug.....................................................................    1      1   seq
.....................acccAguagaaccgagcuugug.....................................................................    3      1   seq
.....................acccuguagaaccgagcuAgug.....................................................................    1      1   seq
.....................acccuguagaaccUagcuugug.....................................................................    1      1   seq
.....................acccuguagaaccgagcuuguU.....................................................................    1863   1   seq
.....................acccuguagaaccgagcuuguUu....................................................................    92     1   seq
.....................acccAguagaaccgagcuugugu....................................................................    1      1   seq
.....................acccuguGgaaccgagcuugugu....................................................................    1      1   seq
.....................acccuguagaaccAagcuugugu....................................................................    5      1   seq
.....................aAccuguagaaccgagcuugugu....................................................................    3      1   seq
.....................acccuguagaaccgagcuuguCu....................................................................    1      1   seq
.....................acccuguagaaccgagcuugugu....................................................................    1159   0   seq
.....................accUuguagaaccgagcuugugu....................................................................    1      1   seq
.....................acAcuguagaaccgagcuugugu....................................................................    1      1   seq
.....................Gcccuguagaaccgagcuugugu....................................................................    15     1   seq
.....................acccuguagaaccUagcuugugu....................................................................    1      1   seq
.....................acccuguagaaccgagcuugugC....................................................................    1      1   seq
.....................acccCguagaaccgagcuugugu....................................................................    1      1   seq
.....................acccuguagaacUgagcuugugu....................................................................    1      1   seq
.....................acccuguagaaccgagcuugugG....................................................................    86     1   seq
.....................accAuguagaaccgagcuugugu....................................................................    1      1   seq
.....................acccuguagaaccgagcuugugA....................................................................    571    1   seq
.....................acccuguagaaccgagcuuguguu...................................................................    33     0   seq
.....................acccuguagaaccgagcuuguUuu...................................................................    3      1   seq
.....................acccuguagaaccgagcuuguguA...................................................................    1      1   seq
.....................acccuguagaaccgagcuuguguuU..................................................................    49     1   seq
.....................acccuguagaaccgagcuuguguuA..................................................................    2      1   seq
```

```
                     Mature                                  Star
ugaaagucacaaugcccauugacccuguagaaccgagcuuguguugacuaauugaaaucacaagccuauucuacaguguacaugguuauugcuaaaacucguccuucuucaa
......................cccuguagaaccgagcuug.......................................................................	1	0	seq
......................cccuguagaaccgagcuugu......................................................................	337	0	seq
......................cccuguagaaccAagcuugu......................................................................	2	1	seq
......................Uccuguagaaccgagcuugu......................................................................	3	1	seq
......................cAcuguagaaccgagcuugu......................................................................	1	1	seq
......................Gccuguagaaccgagcuugu......................................................................	1	1	seq
......................Accuguagaaccgagcuugu......................................................................	2	1	seq
......................cccuguagaaccgagcuuguU.....................................................................	1	1	seq
......................cccuguagaaccgagcuugug.....................................................................	99	0	seq
......................cccuguagaaccgagcuugugA....................................................................	2	1	seq
......................cccuguagaaccgagcuugugu....................................................................	109	0	seq
......................cccuguagaaccgagcuugugG....................................................................	11	1	seq
......................cccuguagaaccgagcuuguguu...................................................................	16	0	seq
......................cccuguagaaccgagcuuguguuU..................................................................	1	1	seq
.......................ccuguagaaccgagcuug.......................................................................	1	0	seq
.......................ccuguagaaccgagcuuUu......................................................................	1	1	seq
.......................ccuguagaaccgagcuugG......................................................................	3	1	seq
.......................ccuguagaaccgagUuugu......................................................................	1	1	seq
.......................ccuguagaaccgagcuugu......................................................................	1002	0	seq
.......................Gcuguagaaccgagcuugu......................................................................	1	1	seq
.......................cUuguagaaccgagcuugu......................................................................	1	1	seq
.......................Acuguagaaccgagcuugu......................................................................	1	1	seq
.......................Ucuguagaaccgagcuugu......................................................................	1	1	seq
.......................ccuguagaaccAagcuugu......................................................................	2	1	seq
.......................ccCguagaaccgagcuugu......................................................................	1	1	seq
.......................ccuguagaaccgagcuuguU.....................................................................	3	1	seq
.......................ccuguagaaccgagcuugug.....................................................................	95	0	seq
.......................ccuguagaaccgagcuugugu....................................................................	80	0	seq
.......................ccuguagaaccgagcuugugA....................................................................	97	1	seq
.......................ccuguagaaccgagcuugugG....................................................................	13	1	seq
........................cuguagaaccgagcuAgu......................................................................	1	1	seq
........................cuguagaaccgagcuugu......................................................................	644	0	seq
........................cuguagGaccgagcuugu......................................................................	1	1	seq
........................cAguagaaccgagcuugu......................................................................	1	1	seq
........................cuguagaaccgagUuugu......................................................................	1	1	seq
........................cuguGgaaccgagcuugu......................................................................	1	1	seq
........................cuguagaaccgagcuugug.....................................................................	30	0	seq
........................cuguagaaccgagcuugugA....................................................................	4	1	seq
........................cuguagaaccgagcuugugu....................................................................	10	0	seq
........................cuguagaaccgagcuuguUuu...................................................................	1	1	seq
........................cuguagaaccgagcuuguguuU..................................................................	1	1	seq
.........................uguagaaccgagcuugug.....................................................................	3	0	seq
.........................uguagaaccgagcuugugu....................................................................	4	0	seq
............................................................caagccuauucuacagug..................................	1	0	seq
............................................................caagccuauucuacaguguac...............................	8	0	seq
............................................................caagccuaAucuacaguguaca..............................	1	1	seq
............................................................caagccuauucuacagAguaca..............................	1	1	seq
............................................................caagccuauucuacaguguaca..............................	146	0	seq
............................................................caagccuauucuacaguguacU..............................	2	1	seq
............................................................caagccuauucuacaguguacaA.............................	2	1	seq
............................................................caagccuauucuacaguguacau.............................	1	0	seq
```
